# Salivary Immune and Metabolic Marker Analysis (SIMMA): A Diagnostic Test to Predict Caries Risk

**DOI:** 10.3390/diagnostics7030038

**Published:** 2017-06-27

**Authors:** Alex Mira, Alejandro Artacho, Anny Camelo-Castillo, Sandra Garcia-Esteban, Aurea Simon-Soro

**Affiliations:** Center for Advanced Research in Public Health, FISABIO Foundation, Valencia 46020, Spain; artacho_ale@gva.es (A.A.); camelo_ann@gva.es (A.C.-C.); garcia_sanest@gva.es (S.G.-E.); simon.aurea@gmail.com (A.S.-S.)

**Keywords:** saliva, dental caries, circadian rhythms, immune system, buffering capacity, pH, adhesion, microorganisms, toothpaste

## Abstract

By using ELISA and colorimetric tests, we have measured 25 compounds in individuals with and without dental caries at different time points of dental biofilm formation and time of the day. We find that some compounds appear to be affected by circadian rhythms, others by dental plaque maturity, and others show constant values during a 24 h period. Using univariate analysis and cross-validation techniques, we have selected six components measured at specific time points that maximize the diagnostic separation of health and disease conditions. Two out of the six selected compounds are related to immune competence, another two to the adhesion capacity of micro-organisms, and another two to acid production or pH buffering. We conclude that, in order to design a robust caries risk test, the time of saliva sampling must be standardized and biomarkers from different categories must be included. The preliminary data shown in this paper provide a proof of principle of a caries risk test based on risk-associated categories. Thus, the test will provide not only a general caries risk assessment, but also the likely biological origin of that risk, namely: immune imbalance, and/or a tendency to adhesion of cariogenic organisms, and/or a lack of acid buffering. When tested longitudinally and validated in larger cohorts, this could open the possibility to develop preventive and personalized treatments.

## 1. Introduction

Dental caries (tooth decay) is the most prevalent chronic disease in the world. Data from the World Health Organization indicate that 80% of the human population suffers or has suffered from it, and it affects over 50% of the population at school age [1]. Dental caries is caused by the acid produced by micro-organisms inhabiting the oral cavity, as a consequence of the fermentation of dietary sugars. This lowers the pH on the tooth surface under a certain threshold, below which the enamel demineralizes, initiating a caries lesion [2]. Once the lesion is cavitated, it is irreversible and the damage can only be restored through clinical intervention, for instance a restoration or a tooth implant.

Although cavities are caused by micro-organisms, dental caries is a multi-factorial disease [3]. Apart from the microbiology, both human-related factors such as immune competence, enamel strength, tooth shape, or saliva buffering effect, and external environmental factors such as diet, oral hygiene, or fluoride exposure have a direct impact on tooth decay rates [4].

Despite its high prevalence and its direct and indirect impact on human health, there are still no effective diagnostic tools to predict dental caries, and therefore dedicate the appropriate personalized measures to prevent the disease. A large effort has been dedicated to study bacterial composition in the oral cavity [5,6,7,8,9], with the aim of developing tests that could relate the presence of acidogenic organisms to caries risk. This has been performed by the direct culturing of specific bacteria [10], or by DNA-based chips that would identify the presence of potentially pathogenic species [11]. The former approach has been clinically applied for years, and includes diagnostic kits for caries risk assessment, focused on culturing the acidogenic mutans streptococci and lactobacilli. However, the bacterial counts of these two bacteria, which have traditionally been considered the main etiological agents of dental caries, have proven to provide limited diagnostic value to predicting disease progression [12]. This can be due to the fact that Streptococcus mutans accounts for less than 1% of the total bacterial community in cavities [17], and that lactobacilli are only found in dentin cavities and not in initial enamel caries lesions, indicating that this species is not involved in caries initiation [13]. Furthermore, the bacterial community within cavities is variable among individuals and cavities [14], hampering the identification of a unique list of pathogenic organisms with predictive value for this polymicrobial disease. In addition, most bacteria-based tests utilize saliva as a sample [9,15], and it has been shown that saliva is not representative of the microbial community at the disease site [14,16,17].

Although caries risk tests based on saliva microbial composition have not been successful in predicting tooth decay, saliva is a powerful sample for measuring overall body health. It is the most easily available and accessible body fluid, and markers expressed in saliva can be used for the diagnosis and patient follow-up of different diseases, including cancer, diabetes, hereditary disorders, and infections [18]. Given that saliva contains many molecules that can directly or indirectly influence oral micro-organisms and their potential cariogenic effect, measuring the levels of appropriate salivary compounds may provide information to predict caries risk [19].

Saliva contains a wealth of substances that are protective of the teeth, and salivary flow has been shown to be a key determinant of protection against tooth decay [20]. In fact, diseases with impaired salivary function, as well as medication reducing saliva production, are frequently accompanied by increased frequencies of tooth decay [21]. There are numerous salivary molecules that could theoretically, and in many cases experimentally, be related to caries propensity, and most of them can be included within three general categories:

1. Molecules related to the Immune System. These include immunoglobulins, antimicrobial peptides, and proteins of the component system, which constitute a protection barrier against oral pathogens [22].
2. Molecules related to the adhesion capacity of micro-organisms. These include structural components of saliva that microorganisms use as targets for sticking to the tooth and forming the dental plaque [23].
3. Molecules related to the acidity of saliva and plaque. These include enzymes that metabolize sugars, acidic compounds that are produced as a consequence of sugar fermentation, and those salivary components that can act as acid neutralizers [24].

A cautionary aspect to be considered when searching for potential caries-associated salivary molecules is that there are several kinds of saliva collection protocols. These include unstimulated, drooling saliva, as well as stimulated saliva after paraffin chewing gum, collection with paper points, oral rinse with saline solution, collection with sterile swabs, or spitting [25], all of which will affect the levels of the different compounds to be measured. In addition, the concentration of salivary components will vary during the day as part of the normal circadian rhythms and changes in salivary flow [26]. As a consequence, for the reliable measure of salivary molecules, a specific sampling protocol and collection time are necessary.

The current work aims at identifying salivary molecules of the three kinds indicated above that could vary in concentration between caries-free and caries-prone individuals, and that could be used as biomarkers of caries risk. We have selected a list of 25 compounds belonging to these three categories that are supported in the literature as potentially or theoretically linked to dental caries, and measured them in caries-free and caries-active adults at four different times during a 24 h period in order to select those molecules and time points with potential diagnostic value. In addition, to predict the caries tendency of an individual, the identification of the underlying reason for the disease’s propensity could allow the design of person-specific preventive treatments in the future. Thus, this preliminary work aims to identify the potential biomarkers of dental caries, and establish the approximate health- and disease-associated concentrations of those compounds.

## 2. Materials and Methods

### 2.1. Donor Selection and Sampling Procedure

An intraoral examination was performed on 20 subjects, gathering information on the presence of caries (including cavitated and non-cavitated enamel lesions as well as dentin caries lesions), plaque deposits (Oral Hygiene Index (OHI)) [27], and the presence of gingival bleeding (Löe and Silness gingival index (GI)) [28], following the recommendations and nomenclature from the World Health Organization [29]. They had not been treated with antibiotics in the three months prior to the study, nor presented with the antecedents of the routine use of oral antiseptics. They were all adults aged 19–39 years. Ten subjects had no history of dental caries (Decayed, Missing and Filled Teeth (DMFT) index = 0), and the other ten had active cavities at the moment of sampling, with or without a history of dental caries (fillings). All of the donors signed an informed consent, and the protocol was approved by the Ethics Committee of the Valencian Public Health Authority (FISABIO-DGSP). The clinical characteristics of subjects are indicated in Table S1.

All of the donors brushed their teeth at 9–10 a.m. using water, to prevent any potential effect of toothpaste on salivary composition and characteristics. Five milliliters of non-stimulated saliva samples were taken by drooling at 30 min, 6, 12, and 24 h after toothbrushing, collecting it in a sterile 50 mL Falcon tube while avoiding spitting or plaque removal by the tongue [30]. The samples were immediately frozen at −80 °C until used. For testing the effect of sugar, drooling saliva samples were collected 10 min after a 1 min oral rinse with a 10% sugar solution.

### 2.2. Quantification of Salivary Immune Components

Enzyme-linked immunosorbent assays (ELISA) and colorimetric tests were performed by duplicate to measure the salivary concentrations of 25 compounds, following the manufacturer’s recommendations. A list of all 25 compounds, including the manufacturer information and the sample dilution used, is shown in Table 1.

### 2.3. Statistical Analysis

We focused on two requirements in terms of usability expectations and model robustness. First, it should include at least two variables from each of the groups “adhesion components”, “acid production/buffering”, and “immune components”. Second, it should be able to provide to the patient two different and complementary risk measurements: on the one hand a univariant-obtained local risk based on the comparison of the patient’s values of selected variables to confidence intervals calculated on caries-free individuals, and on the other hand a multivariant-obtained global risk provided by the overall model. Local-wise analysis would let us design modular therapies focused on one of the groups “adhesion”, “acid production/buffering”, and “immune”, and global-wise analysis would give us an emergency degree for treatment, in order to take those variables out of the confidence intervals and back to normal values.

In order to select the variables included in our model, the Wilcoxon test as implemented in an R environment [31] was performed on each of the candidate variables. The lower the *p*-value provided by the test, the higher is the capability of a variable to distinguish between two groups of samples (Caries-Free and Caries-Active). A non-parametric approach has been adopted to avoid making assumptions about the variables’ distribution. Apart from exhibiting a significant *p*-value, a requirement of a variable to be included in our model is that the confidence intervals (given by lower and upper quantiles) corresponding to the groups of samples Caries and No-Caries do not overlap.

In order to assess the classification accuracy of the variables selected, the k-fold cross-validation technique as implemented in a Galgo R package [32] has been adopted. The dataset was split into k different training and test sets, and the classification accuracy was then defined as the average of the classification accuracies of a model trained on training sets and calculated on the test sets for each of the k splits. Currently, only the set of variables to be included in the model is known; nothing is known about their interactions’ structure. For this reason, we adopted a single-hidden-layer neural network model implemented in a “nnet” R package [33] and offered by Galgo as an unsupervised approach to calculate the classification accuracy of the variables selected.

## 3. Results

### 3.1. Selection of Sampling Time

**Figure 1:**
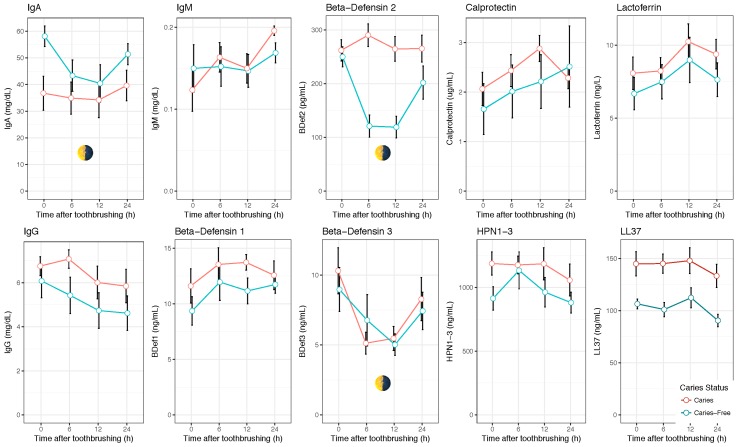
Temporal changes in salivary biomarkers. The graphs show the concentrations (means ± standard error (SE)) of 10 salivary immune components in caries free (*n* = 10) and caries-active (*n* = 10) individuals at four time-points with a 24 h period. Toothbrushing was performed at 9 a.m. with water. Samples were collected at 30 min, 6, 12, and 24 h after toothbrushing. Several compounds increase or decrease in concentration with time after toothbrushing. Other salivary components (marked with a day–night symbol) display a U-shape pattern where the two morning samples have similar concentrations, suggesting that they are influenced by circadian rhythms. Potential biomarkers include LL37, which appears to discriminate between caries-free and caries-active groups at all time points, or β-defensin 2, which shows large differences between caries-free and caries-active individuals only in the afternoon and evening samples.

An initial test was performed with 10 compounds (IgA, IgG, IgM, α-defensin 1–3, β-defensin 1, β-defensin 2, β-defensin 3, LL-37, Lactoferrin, and Calprotectin), which were measured in saliva samples taken at 0.5, 6, 12, and 24 h after toothbrushing. These sampling points corresponded to 9–10 a.m., 3–4 p.m., 9–10 p.m., and 9–10 a.m. the next morning, respectively. By using these four moments, the potential effect of daily rhythms, as well as the effect of dental plaque maturity, could be evaluated. A period of 30 min after toothbrushing was chosen, to allow for the stabilization of the salivary concentrations that could be altered due to the mechanical tissue abrasion. Important concentration changes were observed across time for most compounds, indicating that the salivary levels of these proteins are not constant (Figure 1). The trends were, however, different depending on the compound. IgG, for instance, showed a decrease in salivary concentration from the time of toothbrushing, whereas Calprotectin displayed an increase through time. This suggests that the time of toothbrushing could have an effect on the salivary concentrations of some compounds. For some proteins, such as IgA, β-defensin 2, or β-defensin 3, a clear U-shape pattern was observed for caries-free individuals, where the concentrations decreased during the afternoon and night but were higher in the two morning samples, suggesting an influence of circadian daily rhythms (the *p*-values for the comparison between the morning and afternoon samples were 0.019 for IgA, 0.0005 for β-defensin 2, and 0.019 for β-defensin 3; the *p*-values for the comparison between the two morning samples were, respectively, 0.11, 0.35, and 0.58 (Wilcox test)). Interestingly, the salivary concentrations of IgA and β-defensin 2 in caries-active individuals appeared to be constant through time, as a consequence of which the levels of these two compounds in the afternoon and evening were significantly different between the caries-active and caries-free groups (Table 2). Thus, the molecules that could be good biomarkers of the disease at a given time may not discriminate between healthy and caries-risk individuals at another time point. We hypothesize that this can be one of the reasons why the results of salivary tests which do not specify a sampling time may lack accuracy or consistency.

Given that 12 and 24 h after toothbrushing will not represent a comfortable and reliable sampling time for clinical use, and that the sampling has ideally to be adjusted to a clinic’s opening hours, the morning and afternoon timepoints, corresponding to 0.5 and 6 h after toothbrushing, were considered for further study, and the measurements of all 25 salivary components were performed at these two timepoints. The measured concentrations of the selected 25 components from the three categories for caries-free and caries-active individuals are indicated in Figure S1A,C,E (values at 0.5 h, morning sample) and Figure S1B,D,F (values at 6 h, afternoon sample).

### 3.2. Selection of Caries-Associated Biomarkers

**Figure 2:**
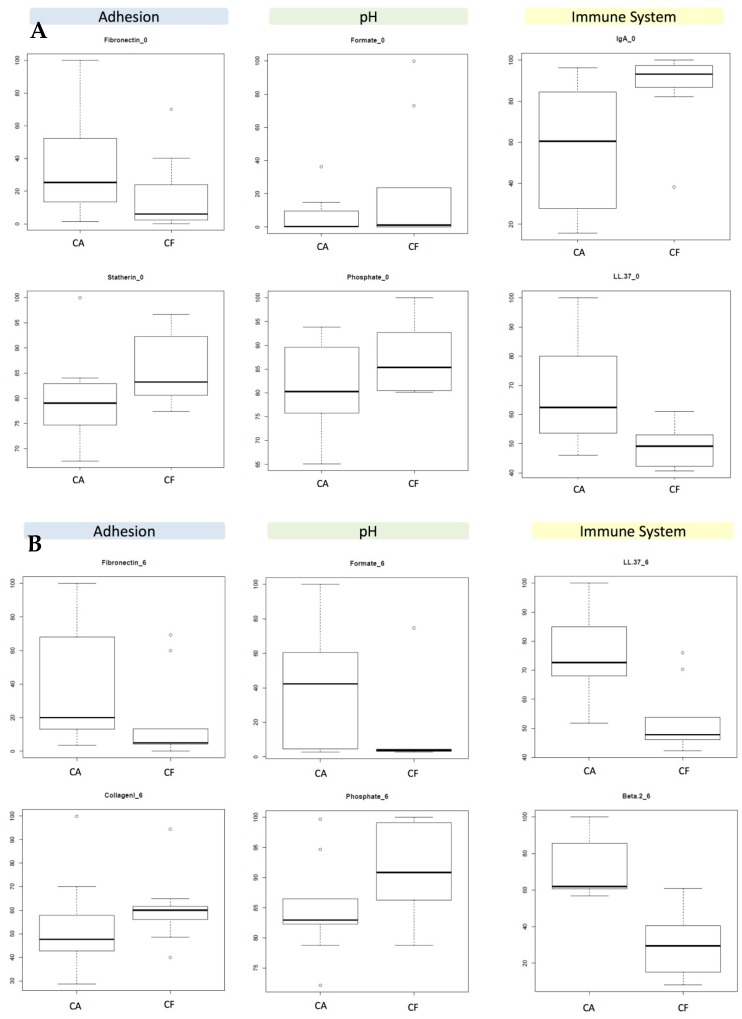
Concentrations of potential salivary biomarkers of dental caries. The boxplots represent normalized median and interquartile ranges for 10 caries-active (CA) and 10 caries-free (CF) individuals in 6 salivary components with potential diagnostic value at two time-points: (**A**) 0.5 h after tooth brushing (morning sample); and (**B**) 6 h after tooth brushing (afternoon sample.

The medians and upper/lower quartiles of all of the salivary components in caries-free and caries-active individuals are shown for the 0.5 h measurements (Figure S1A,C,E and for the 6 h measurements (Figure S1B,D,F) for immune molecules, adhesion molecules, and acid/buffering components. Wilcox univariate tests were performed to compare the values between individuals with and without caries (Table 2). As can be observed, few of the measured variables in fact have diagnostic value, even if they belong to the same category. The medians and interquartile ranges of the two components from each category with the best discriminating capacity are shown in Figure 2.

**Figure 3:**
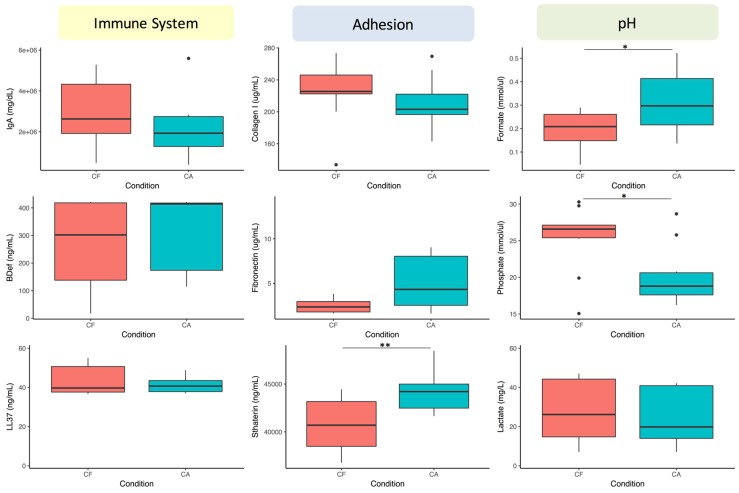
Effect of sugar rinse on salivary concentrations of potential biomarkers. Unstimulated saliva samples were collected 10 min after a 1 min rinse with a 10% sucrose solution performed between 9–10 a.m. Relative to the morning samples in Table 2 and Figure 2, Phosphate and Formate increase their discriminating power. The boxplots show the median and interquartile range values. Asterisks indicate statistical significance (Wilcox rank sum test).

The data show that immune components are the ones that better discriminate between healthy and diseased individuals. This suggests an important role for immune competence in the risk of developing caries. At 6 h after tooth brushing (afternoon sample), several components of each of the three categories were different between the two patient groups, whereas at 0.5 h (morning sample) no differences were found in the acidic component category. The latter could be due to the fact that these metabolites are mainly produced after dietary carbohydrate fermentation, and are more readily measured at 6 h (after lunch in our sampling schedule). In order to test this possibility, the same test was repeated in the morning but 10 min after a 1 min rinse with a 10% sugar solution. The results show an improvement in the discriminatory power of Statherin and of compounds in the pH buffering category, specifically Formate and Phosphate (Figure 3). Curiously, Lactate’s discriminatory power did not improve after the sugar rinse. The biomarker concentrations in the other two categories were affected by the sugar rinse, and although the overall tendency for the selected biomarkers in the adhesion and immune categories was maintained, the difference between caries-active and caries-free individuals was significant only for Statherin.

Thus, based on the *p*-values from the univariate analyses (Table 2) and the lack of overlap between the data dispersion boxes (Figure S1), the following salivary metabolites are selected to provide discrimination value between healthy and caries-active individuals:

1. At 6 h:
  - Immune molecules: β-defensin 2 and LL-37.
  - Adhesion molecules: Collagen I and Fibronectin.
  - pH components: Formate and Phosphate.
2. At 0.5 h:
  - Immune molecules: LL-37 and IgA.
  - Adhesion molecules: Statherin and Fibronectin (Statherin only if saliva is collected after a sugary solution rinse).
  - pH components: Phosphate and Lactate (Phosphate and Formate if saliva is collected after a sugary solution rinse).

**Figure 4:**
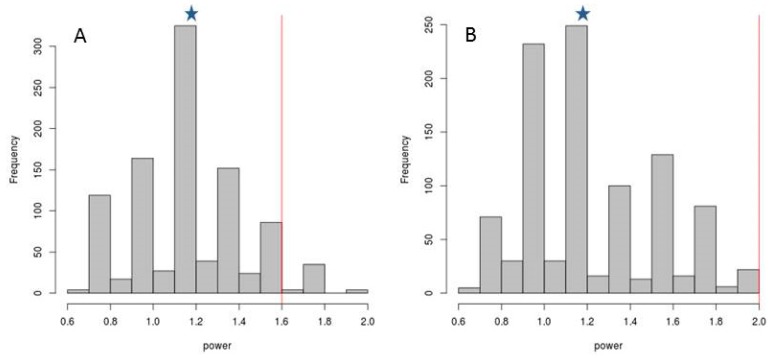
Statistical classification power of salivary components. The *X*-axis in the frequency histograms indicate the classification power of 1000 combinations of 6 components randomly selected from the 25 compounds measured in saliva in the current manuscript, including the 6 components selected as the best biomarkers in the present work, for samples taken at (**A**) 0.5 h (morning sample) and (**B**) 6 h (afternoon sample) after brushing teeth. Values in the *Y*-axis represent the number of random combinations for each statistical power category. The median classification power of the six randomly selected compounds was 1.2 at both 0.5 and 6 h (marked with asterisks), whereas the median classification power of the selected biomarkers (marked with red vertical lines) was 1.6 and 2.0 at 0.5 and 6 h after tooth brushing, respectively. Power = (proportion of correct classification of caries individuals (CA)) + (proportion of correct classification of caries-free individuals (NOCA)), wherein said proportions were estimated using a 50-fold cross-validation approach.

In order to test whether any combination of concentrations of any of the aforementioned 25 compounds present in saliva may improve caries risk prediction, the statistical classification power was compared between the six variables selected above (those with the best *p*-values) and 1000 random selections of variables. Power may be defined as (proportion of correct classification of caries individuals (CA)) + (proportion of correct classification of caries-free individuals (NOCA)). Thus, the maximum classification power value is 2. When the potential biomarkers of caries risk were randomly selected in groups of six, the combinations did not improve the diagnostic value provided by the six selected compounds, neither at 0.5 h after brushing teeth (Figure 4A) or at 6 h (Figure 4B). Specifically, the median classification power of the six randomly selected compounds was 1.2 at both 0.5 and 6 h. Thus, it may be concluded that the six selected variables are those that maximize the diagnostic value of all of the measured biomarkers, especially in the afternoon samples.

The classification accuracy of the selected variables was measured by a cross-validation unsupervised approach [33], indicating that, on average, 98% of the caries individuals are detected by the test at both timepoints. Increasing the number of variables from the six selected above to the eight most significantly different compounds did not improve this percentage. The cross-validation technique using the Galgo method for data sampled at 6 h afforded a sensitivity of 98% and a specificity of 88%. In other words, almost 100% of subjects with caries are classified correctly, whereas only 12% of subjects without caries are falsely assigned to the high caries risk group. It is possible that the 12% of false positives arise within the group of subjects without caries because these subjects in fact have high caries risk, but have not clinically developed this condition due to, for example, the quality of their diet and/or oral hygiene. Unfortunately, we did not collect diet data, and the validity of this hypothesis should be tested with larger sample sizes, especially in longitudinal studies.

## 4. Discussion

**Figure 5:**
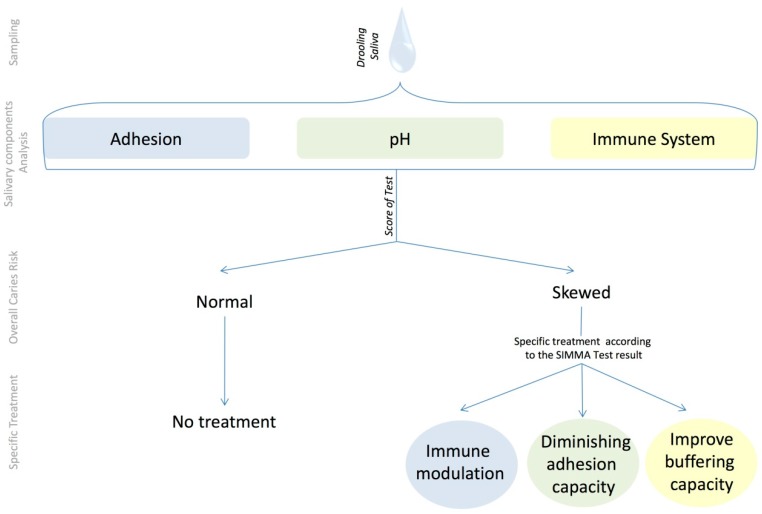
Rationale of the Salivary Immune and Metabolic Marker Analysis (SIMMA) test. An unstimulated saliva sample is used to measure different compounds belonging to three functional categories, and compare their concentrations to those or healthy, caries-free individuals from the same age. A skewed concentration for any of those biomarkers is considered to represent an imbalance in the corresponding category, opening possibilities for individual-specific preventive measures.

Based on the above preliminary data, a Salivary Immune and Metabolic Marker Analysis test (SIMMA test) is proposed, which is based on measuring the salivary values from an individual at a given time point of six selected compounds, two of which are related to immune competence, another two to the adhesion capacity of micro-organisms, and another two to the acid production and buffering capacity. Those values are then compared to the reference values obtained from a healthy population of a similar age, and the concentrations falling outside the healthy range are indicative of caries risk due to an imbalance in the corresponding category. Thus, the test will provide not only a general caries risk assessment, but also the likely biological origin of that risk, namely: immune imbalance, and/or a tendency to adhesion of cariogenic organisms, and/or a lack of acid buffering. Based on the SIMMA test outcome, a preventive, personalized treatment will be possible, directed towards one or more of the following goals: (i) immune modulation to select a non-cariogenic oral biofilm, which could be achieved, for example, by probiotic bacteria that have been shown to stimulate antibody production (see [34] for a recent review); (ii) diminishing the adhesion capacity of a cariogenic biofilm, which could be achieved by specific anti-adherent molecules (see for example [35]) added to daily dental hygiene products; and (iii) improving buffering capacity, which could be achieved by stimulating salivary flow through chewing or by the addition of buffering molecules or prebiotic compounds that stimulate ammonia production (see for example [36]) to daily dental hygiene products. A flow chart of the SIMMA test, its rationale, and applications is shown in Figure 5.

Our data also underline the importance of standardizing sampling time, because some molecules with potential diagnostic value are subject to daily rhythms. Although we did not measure salivary flow in our samples, observed daily changes in the levels of some compounds must partly be due to salivary flow, which is known to follow a circadian rhythm [26], where lower saliva levels in the morning would tend to elevate solute concentrations. Thus, if sampling time is not taken into account, an individual salivary biomarker may have more predictive power when the data are normalized with salivary flow rates or total protein concentration (a flow rate dependent parameter).

The univariate analysis determines the individual salivary compounds that, once measured and compared to the healthy reference values, will suggest the appropriate treatment to prevent the appearance of caries. In addition to this, it must be kept in mind that the combination of measurements will be more informative and sensitive than individual ones. For instance, an individual may present normal values for a given compound but have out-of-range values for another. This is one of the reasons why tests based on individual variables will likely lack the sensitivity to detect the risk of caries. In addition, not only the values of each compound but also the interaction among them may provide information about an individual’s caries risk. Thus, combining the values of all of the selected compounds measured in a multivariate analysis should also be performed to provide an overall caries risk value. This overall value will inform the clinician about the general tendency of the patient to develop caries. In practice, the number of out-of-range compounds could also serve in the clinic as a measure of the caries risk in a patient, and therefore their treatment’s urgency. This information could also serve to determine individuals at risk, where the frequency of visits and the type of interventions can be adapted to reduce the probability of future caries development [37,38].

## 5. Conclusions

In conclusion, a test based on the selection of biomarkers from different risk-associated categories will provide an overall caries risk value and a list of salivary components that show skewed values. The test should be performed at a specific timepoint and time since toothbrushing, given that both factors, especially daily rhythms, affect salivary compounds’ concentration. If urine or blood tests have to be performed under specific conditions or at timepoints for determining the health boundaries of biomarkers, it is not unreasonable to assume that the same standardization has to be achieved with salivary tests. The functional category to which those skewed components belong may provide a putative prevention treatment to restore values to the healthy range.

A limitation of the current study is clearly the small sample size. Nevertheless, the preliminary data shown in this paper provide a proof of principle of a caries risk test based on risk-associated categories. The specific boundaries of health and disease in the concentrations of the different biomarkers are likely to be age-specific, and should be quantified in study groups of different ages, especially children, which is the group in which preventive strategies are most fruitful. Although not shown in this paper, we measured salivary pH and pH buffering capacity in the same samples using commercial kits, but these basic measurements failed to discriminate between caries-free and caries-active individuals. However, we did not measure other variables normally used for caries risk assessment, such as the salivary levels of cariogenic organisms, or dietary habits. Thus, the test proposed in this paper should be compared with the methods that are currently accepted in the assessment of caries risk (see, for example, [37,38]). Once the appropriate biomarkers have been selected, the SIMMA test should be transformed from the current laboratory measurements into a ready-to-use kit based on reactive strips, where out-of-range values for one or two biomarkers per category can be easily and quickly visualized without the need for laboratory equipment. The use of diagnostic strips has been successfully applied to determine the risk of periodontal disease in adolescents based on the levels of the human matrix metalloproteinase MMP-8 [39]. Similarly, the development of point-of-care diagnostic strips could be instrumental for an application of caries risk tests at a community level. Especially relevant would be the application of caries risk assessment in children, in order to determine those individuals at high risk where preventive measures could be implemented. Some of those, like the sealing of pits and fissures for caries prevention, would be too costly and unnecessary to perform on all children, and a test able to select high-risk patients would be extremely helpful [40]. In private clinical practice, the identification of high-risk individuals, and especially the putative cause of the risk, would provide the dentist with valuable information to personalize the treatment, as well as to establish the timing of visits. The development of caries risk assessment methods in order to achieve personalized, “precision” dentistry is both desirable and achievable, but several conceptual and analytical mistakes have been highlighted, including the application of population-level variables to individuals or the use of inappropriate modeling [41]. The data presented in this paper show an association of some salivary components with an existing caries status. When appropriate health thresholds are established for the different biomarkers, longitudinal studies will determine whether those compounds are not only disease-associated, but have also a predictive value.

## Figures and Tables

**Table 1:** Salivary components measured in the current work and manufacturer of the kit used for determining their concentrations.

Category	Salivary Compound	Manufacturer	Sample Dilution
Immune System	IgA	Assaypro	1:1000
	IgG	Assaypro	1:10
	IgM	Assaypro	1
	Alpha-defensin 1-3	Assaypro	1:10
	β-defensin 1	Sun Red	1
	β-defensin 2	Sun Red	1
	β-defensin 3	Sun Red	1
	Cathelicidin LL-37	Assaypro	1:200
	Lactoferrin	Assaypro	1:20
	Calprotectin	Assaypro	1
	Lysozyme	Assaypro	1:8000
	C3a	Sun Red	1:10
Adhesion	PRB1	Sun Red	1:10
	Statherin	Sun Red	1:200
	Collagen type I	Sun Red	1
	Mucin C7	Sun Red	1:50
	Mucin C5B	Sun Red	1:100
	Alpha-2 macroglobulin	Assaypro	1:4
	Fibronectin	Assaypro	1:20
pH	Lactate	BioVision	1:20
	Formate	BioVision	1:5
	Calcium	BioVision	1
	Phosphate	BioVision	1:200
	Urea	BioVision	1:25
	Alpha-amylase	Biovision	1:10

**Table 2:** Statistical significance for the comparison of 25 measured salivary compounds between caries-free and caries-active individuals at two different times after tooth brushing (performed at 9 a.m.).

Category	Compound	0.5 h	6 h
Immune System	Cathelicidin LL-37	0.007	0.002
	IgA	0.007	0.353
	α-Defensin 1-3	0.105	0.853
	IgM	0.326	1.000
	β-Defensin 1	0.393	0.796
	Lactoferrin	0.393	1.000
	C3a	0.393	0.315
	Calprotectin	0,405	0.326
	β-Defensin 3	0.520	0.970
	IgG	0.520	0.121
	β-Defensin 2	0.971	7.57 × 10^−5^
	Lysozyme	0.796	0.280
Adhesion	Statherin	0.063	0.247
	Fibronectin	0.123	0.121
	Mucin 5B	0.218	0.684
	Mucin 7	0.247	0.571
	Collagen I	0.273	0.104
	Alpha-2 Macroglobulin	0.393	0.796
	PRB1	0.912	0.190
pH	Phosphate	0.140	0.054
	Lactate	0.353	0.123
	Urea	0.393	0.393
	Calcium	0.631	0.315
	Formate	0.853	0.029

## Supplementary Materials

The following are available online at www.mdpi.com/2075-4418/07/3/38/s1.
