# Supplementary material for: Salivary Immune and Metabolic Marker Analysis (SIMMA): A Diagnostic Test to Predict Caries Risk
Source: Diagnostics (Basel). 2017 Jun 27;7(3):38. doi: 10.3390/diagnostics7030038 (PMC5617938; doi:10.3390/diagnostics7030038)
Supplement: Supplementary file 1 [file diagnostics-07-00038-s001.zip › diagnostics-195057 supplementary/Figure S1.pdf]

Figure S1: Salivary concentrations of the 25 measured compounds.

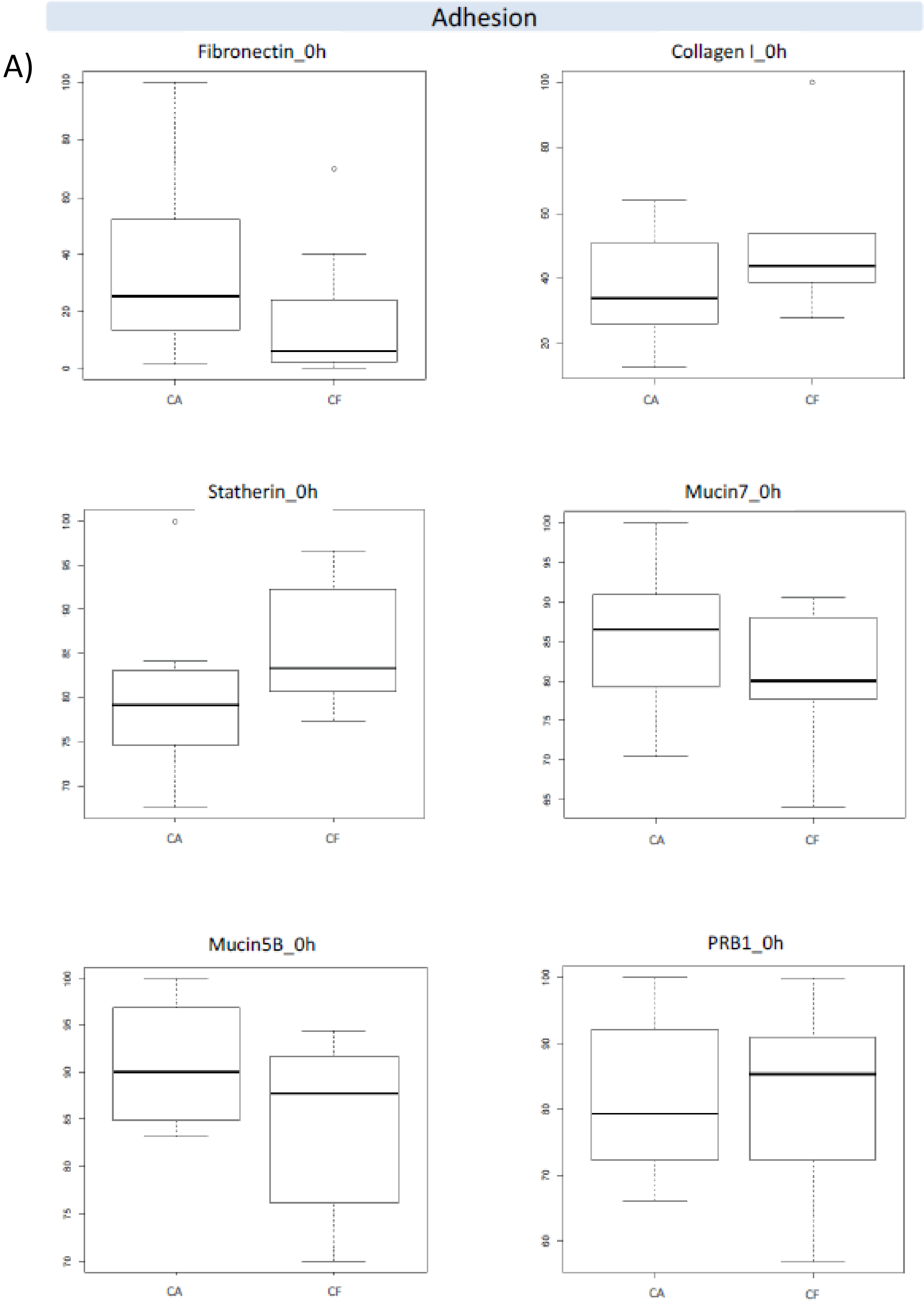

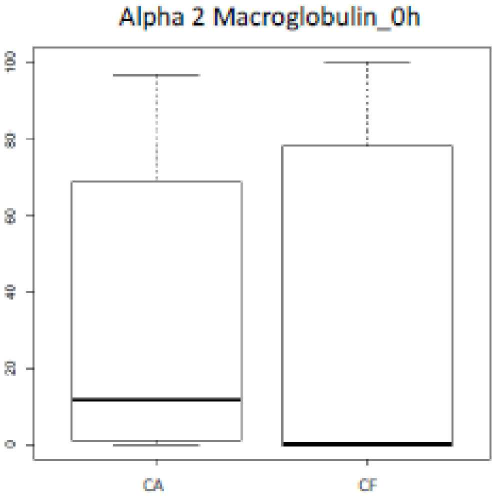
Alpha 2 Macroglobulin_0h
100
80
60
40
20
0
CA
CF

B)

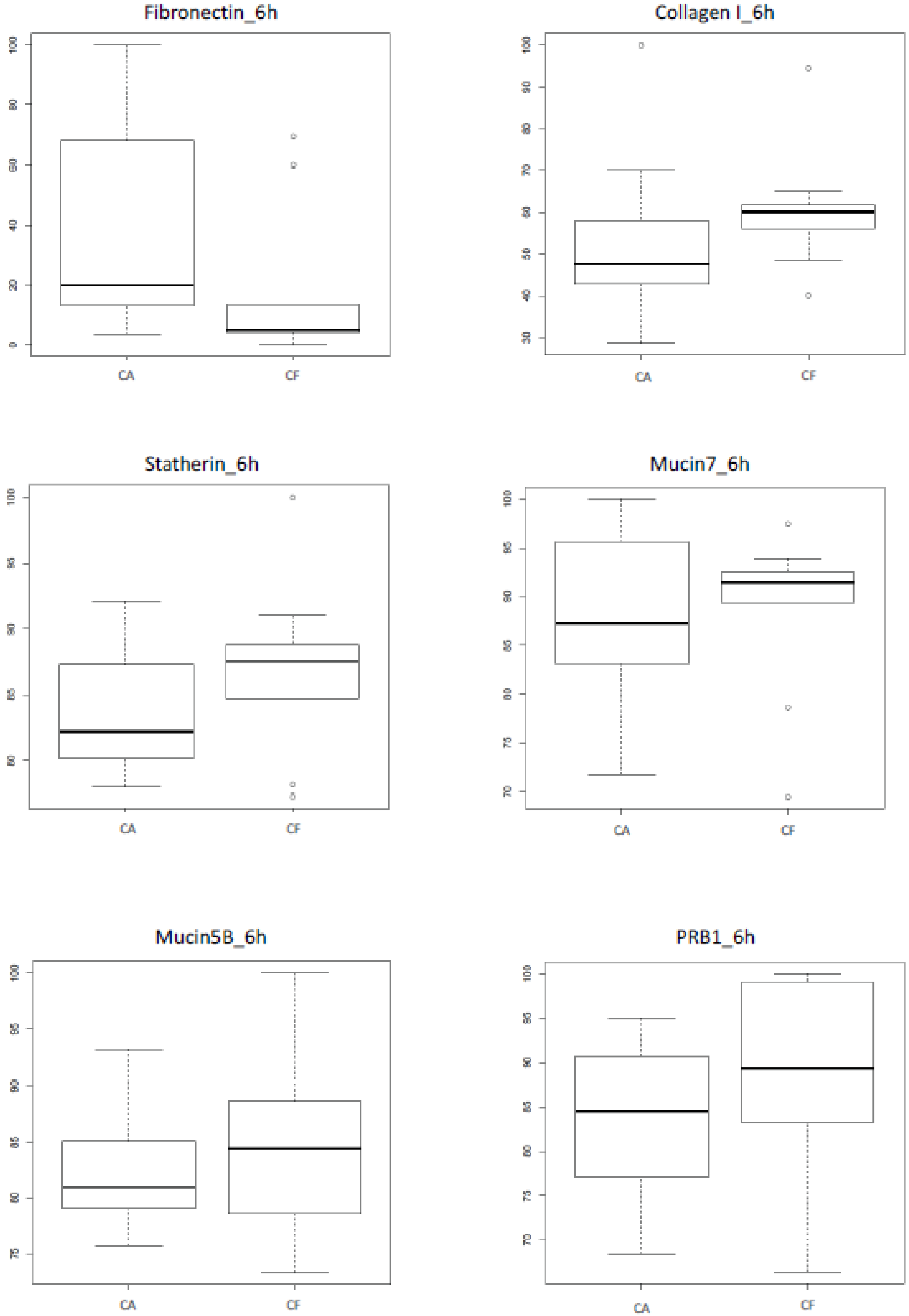
Fibronectin_6h
CA
CF
Collagen I_6h
CA
CF
Statherin_6h
CA
CF
Mucin7_6h
CA
CF
Mucin5B_6h
CA
CF
PRB1_6h
CA
CF

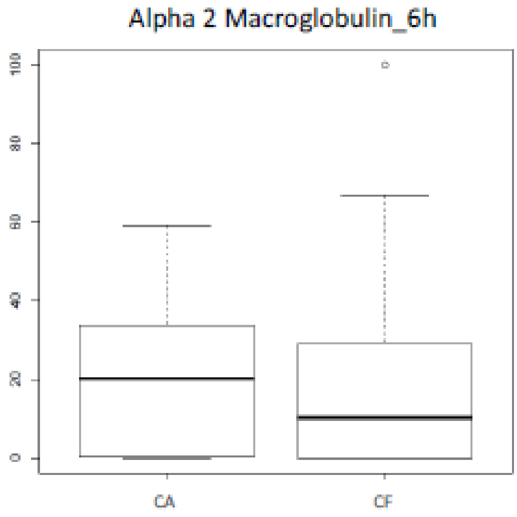

Alpha 2 Macroglobulin_6h
0
20
40
60
80
100
CA
CF

C)

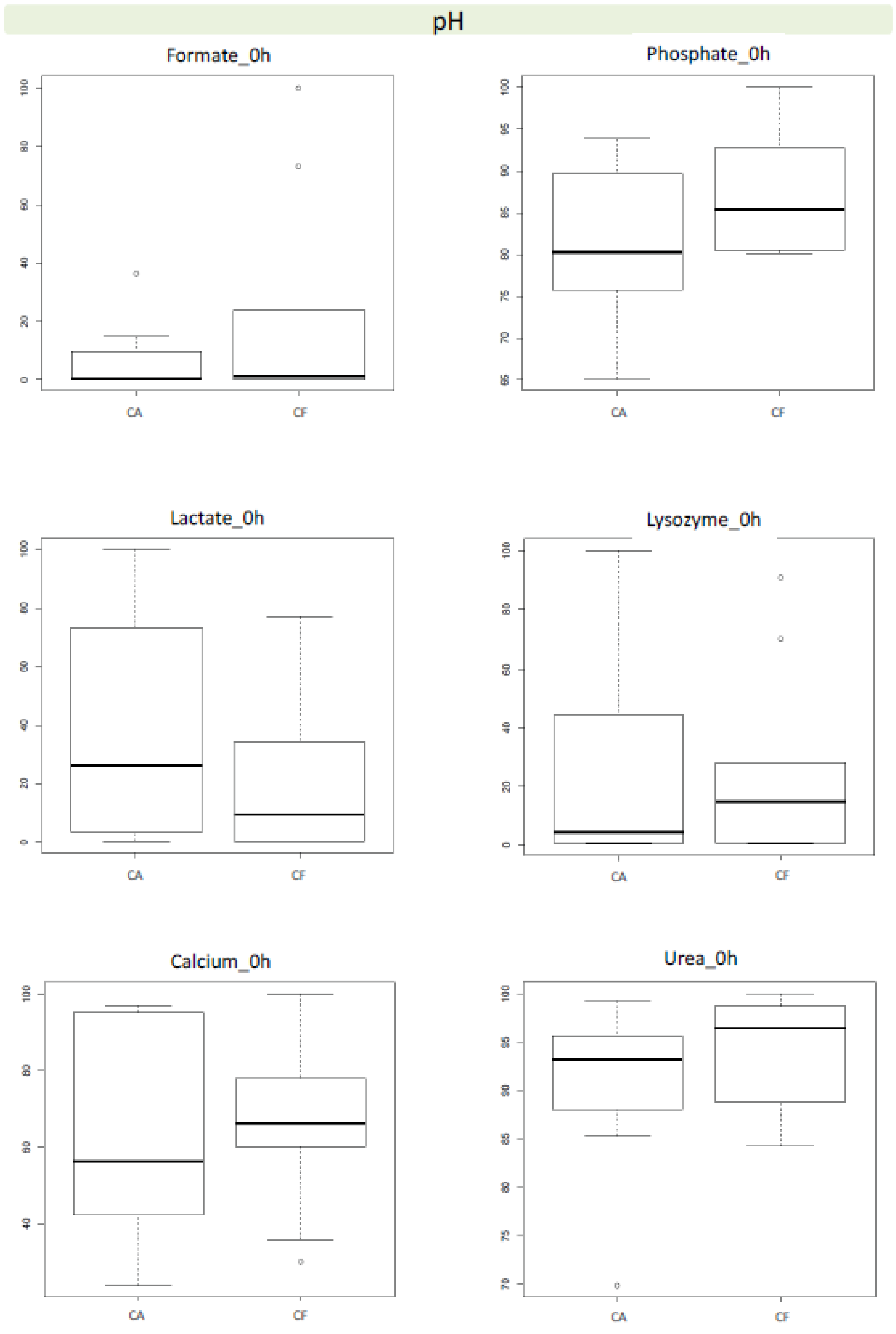
pH
Formate_0h
CA
CF
Phosphate_0h
CA
CF
Lactate_0h
CA
CF
Lysozyme_0h
CA
CF
Calcium_0h
CA
CF
Urea_0h
CA
CF

D)

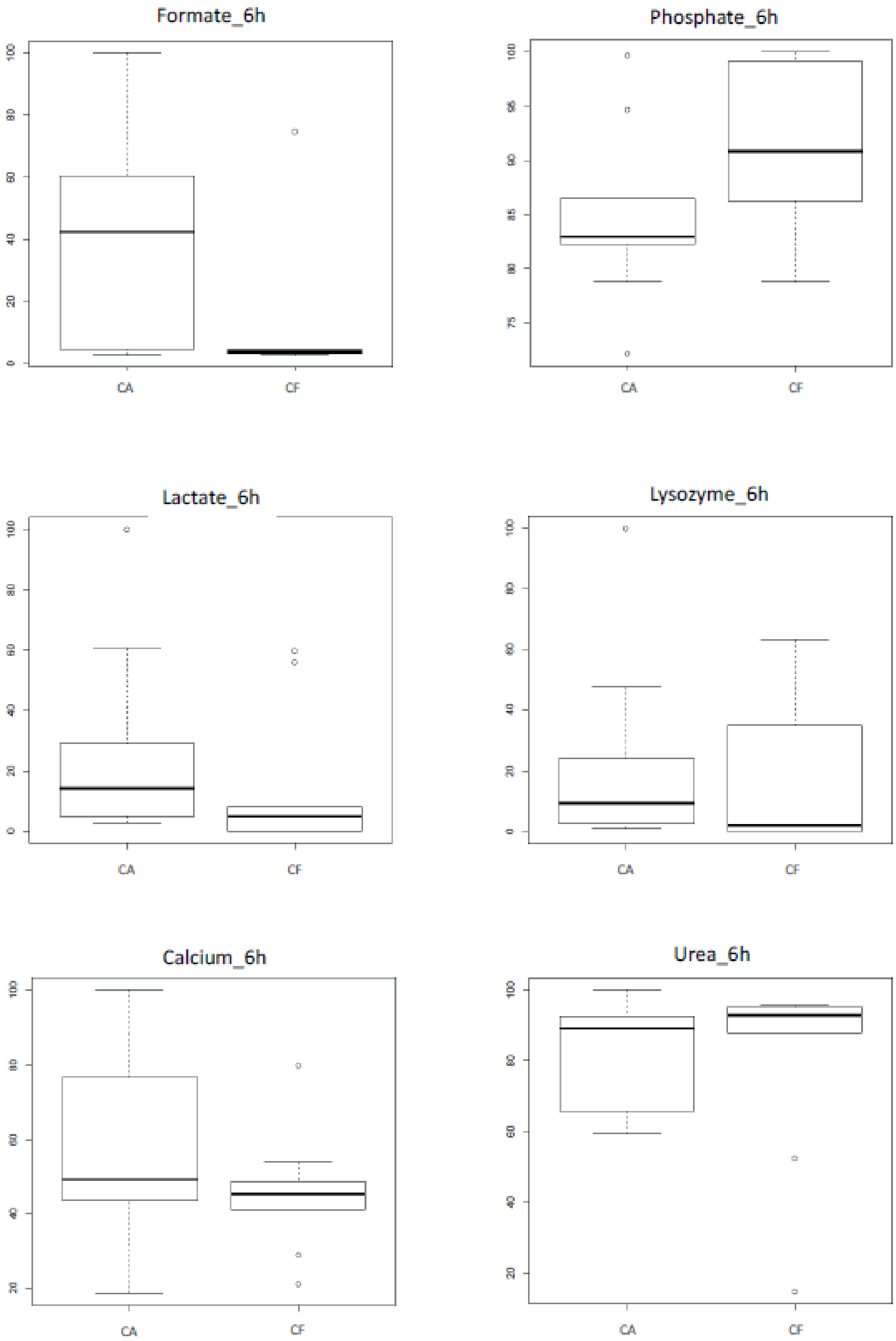

Formate_6h
CA
CF
Phosphate_6h
CA
CF
Lactate_6h
CA
CF
Lysozyme_6h
CA
CF
Calcium_6h
CA
CF
Urea_6h
CA
CF

E)

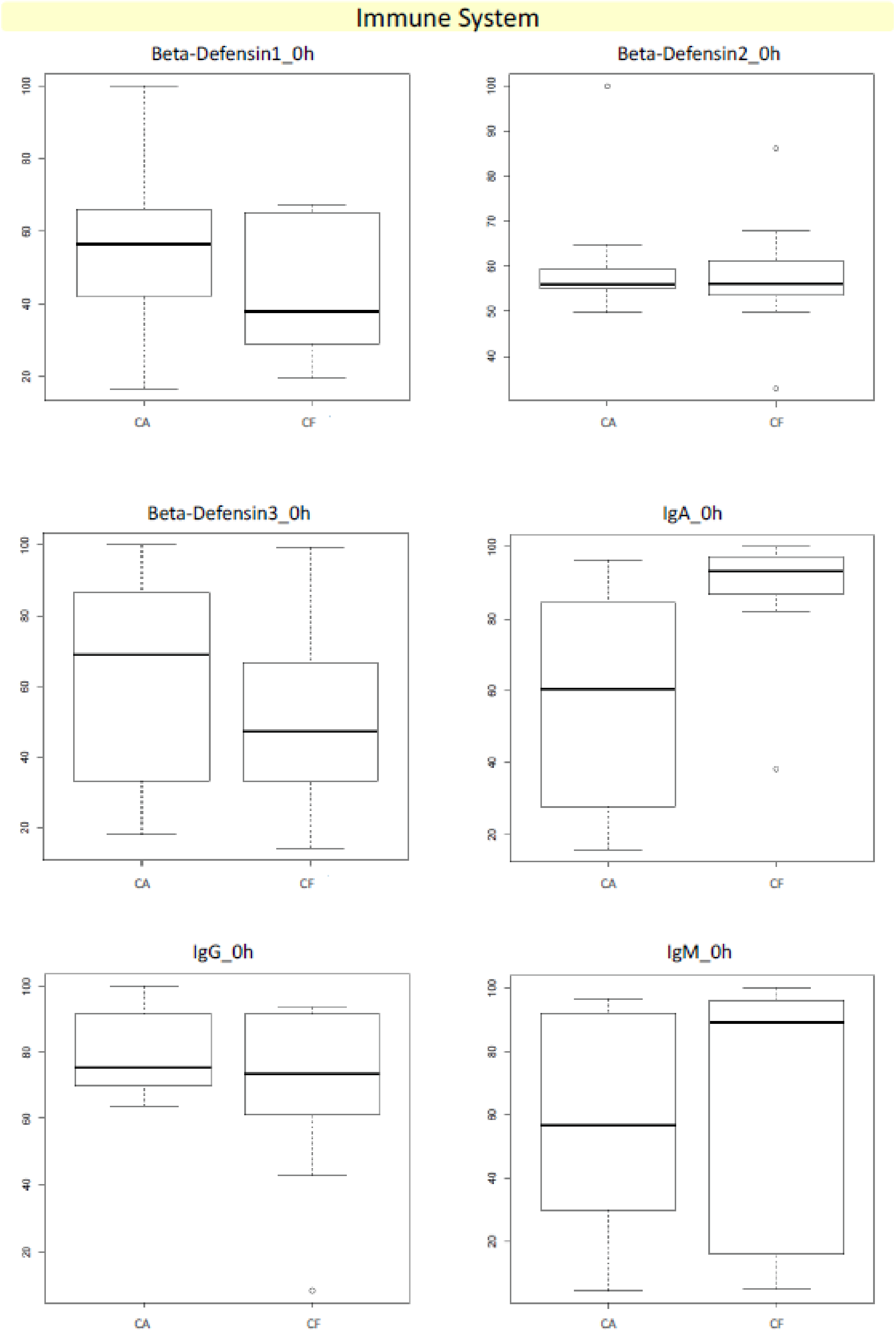
Immune System
Beta-Defensin1_0h
CA
CF
Beta-Defensin2_0h
CA
CF
Beta-Defensin3_0h
CA
CF
IgA_0h
CA
CF
IgG_0h
CA
CF
IgM_0h
CA
CF

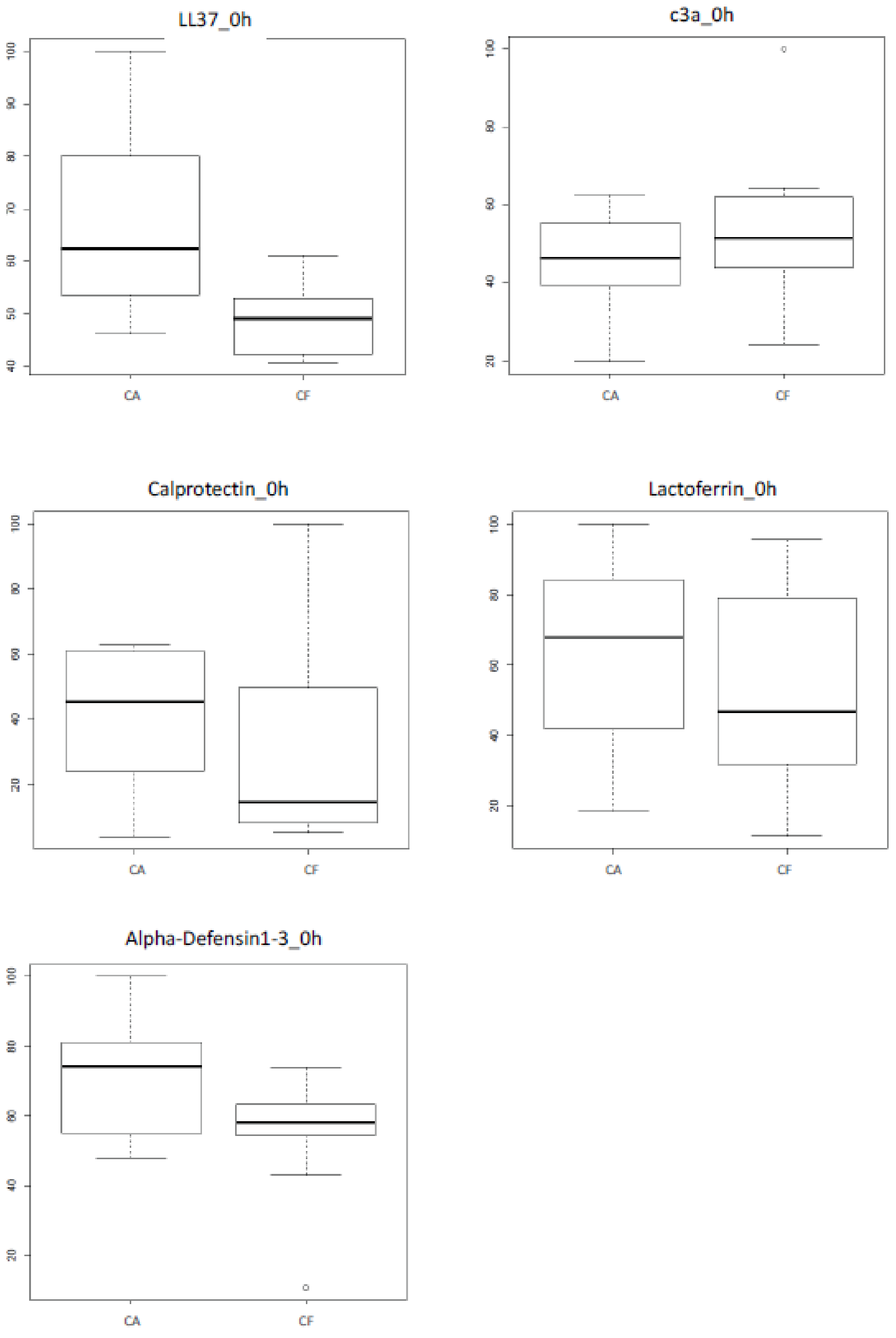
LL37_0h
CA
CF
c3a_0h
CA
CF
Calprotectin_0h
CA
CF
Lactoferrin_0h
CA
CF
Alpha-Defensin1-3_0h
CA
CF

F)

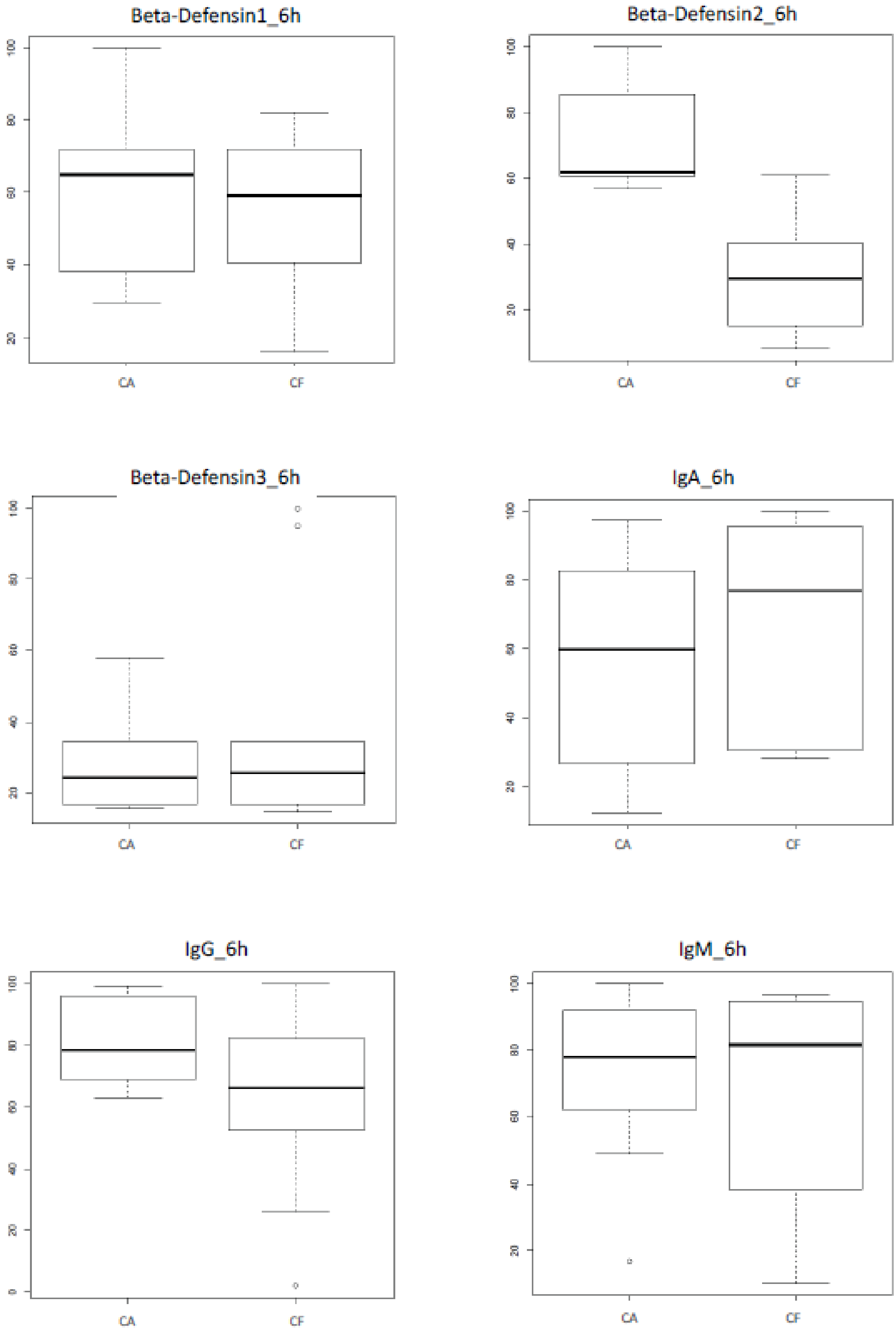
Beta-Defensin1_6h
CA
CF
Beta-Defensin2_6h
CA
CF
Beta-Defensin3_6h
CA
CF
IgA_6h
CA
CF
IgG_6h
CA
CF
IgM_6h
CA
CF

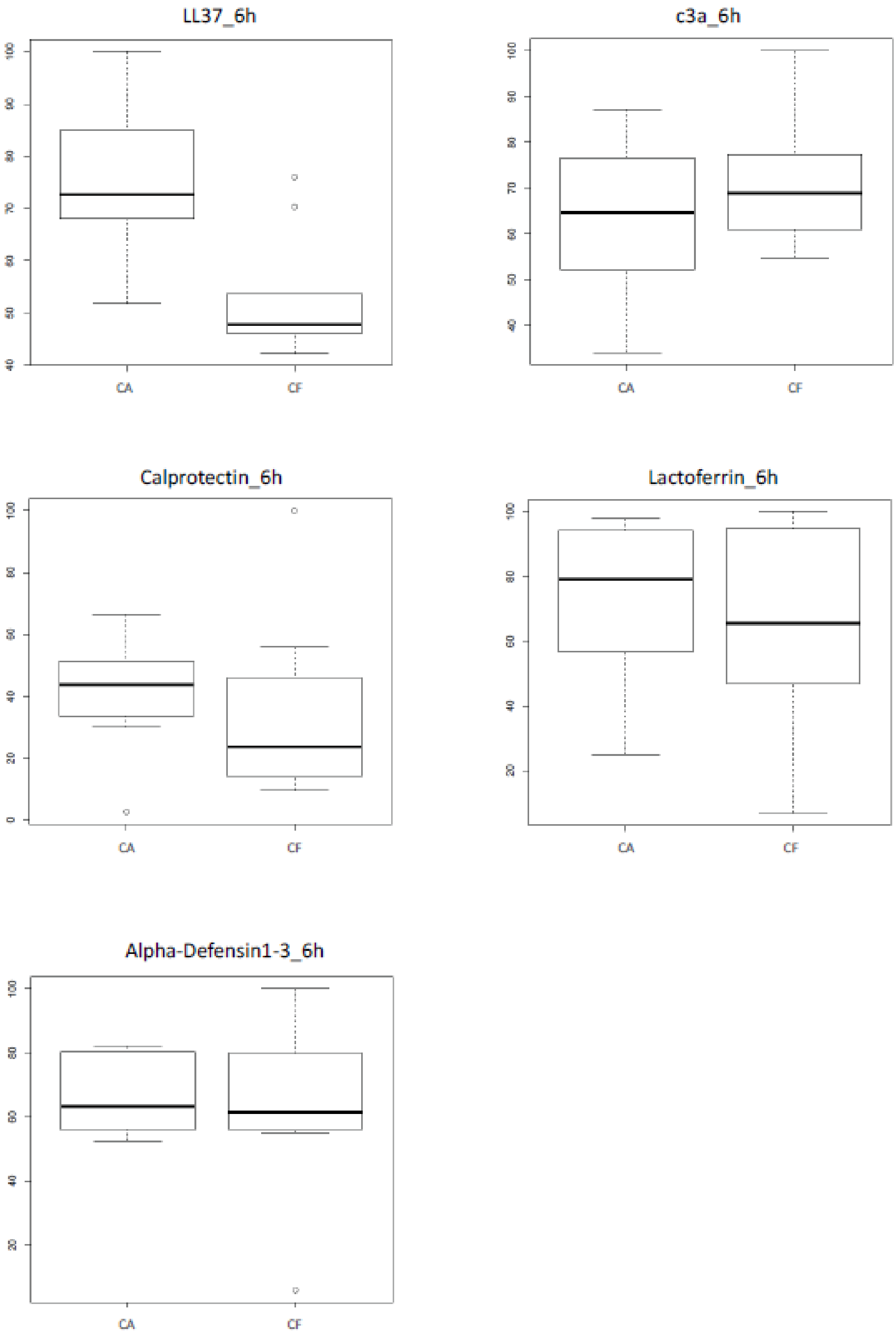
LL37_6h
CA
CF
c3a_6h
CA
CF
Calprotectin_6h
CA
CF
Lactoferrin_6h
CA
CF
Alpha-Defensin1-3_6h
CA
CF
