# Supplementary material for: Salivary Immune and Metabolic Marker Analysis (SIMMA): A Diagnostic Test to Predict Caries Risk
Source: Diagnostics (Basel). 2017 Jun 27;7(3):38. doi: 10.3390/diagnostics7030038 (PMC5617938; doi:10.3390/diagnostics7030038)
Supplement: Supplementary file 1 [file diagnostics-07-00038-s001.zip › diagnostics-195057 supplementary/Supplementary Table S1.pdf]

Supplementary Table S1. Clinical characteristics of studied patients.

Sample	Sex	Age	OHI[1]	GI[2]	Decayed	Filled	Missing
NOCA.TV01	Male	24	0	1	0	0	0
NOCA.TV02	Male	39	0	0	0	0	0
NOCA.TV04	Male	25	0	1	0	0	0
NOCA.TV05	Female	27	0	0	0	0	0
NOCA.TV13	Male	26	0	1	0	0	0
NOCA.TV07	Male	19	3	2	0	0	0
NOCA.TV08	Female	31	1	0	0	0	0
NOCA.TV09	Male	29	1	0	0	0	0
NOCA.TV10	Female	39	1	0	0	0	0
NOCA.TV11	Male	37	2	1	0	0	0
CATV.02	Male	29	0	0	1	6	0
CATV.01	Male	34	0	0	3	5	0
CATV.04	Female	25	0	0	3	0	0
CATV.05	Male	38	1	1	5	14	0
CATV.06	Male	33	1	1	4	0	0
CATV.03	Male	37	1	1	1	4	0
CATV.08	Female	30	0	0	3	12	0
CATV.09	Female	27	1	1	2	2	4*
CATV.10	Female	25	0	0	8	8	0
CATV.07	Male	27	3	3	9	12	0

[1] Oral Hygiene Index: 0.- No visible plaque. 1.- No visible plaque, but adheres to probe. 2.- Low to moderate thickness plaque. 3.- Thick and abundant plaque

[2] Gingival Index: 0.- Healthy. 1.- Mild inflamation. 2.- Redness and/or induced bleeding. 3.- Spontaneous bleeding

*removed for orthodoncic reasons
